# The mechanism of chronic intracellular infection with *Brucella* spp.

**DOI:** 10.3389/fcimb.2023.1129172

**Published:** 2023-04-18

**Authors:** Xiaoyi Guo, Hui Zeng, Mengjuan Li, Yu Xiao, Guojing Gu, Zhenhui Song, Xuehong Shuai, Jianhua Guo, Qingzhou Huang, Bo Zhou, Yuefeng Chu, Hanwei Jiao

**Affiliations:** ^1^ The College of Veterinary Medicine, Southwest University, Chongqing, China; ^2^ Changchun Veterinary Research Institute, Chinese Academy of Agricultural Sciences, Changchun, Jilin, China; ^3^ State Key Laboratory of Veterinary Etiological Biology, College of Veterinary Medicine, Lanzhou University, Lanzhou Veterinary Research Institute, Chinese Academy of Agricultural Sciences, Lanzhou, Gansu, China; ^4^ The Immunology Research Center, Medical Research Institute, Southwest University, Chongqing, China

**Keywords:** *Brucella*, chronic infections, autophagy, metabolism, apoptosis

## Abstract

Globally, brucellosis is a widespread zoonotic disease. It is prevalent in more than 170 countries and regions. It mostly damages an animal’s reproductive system and causes extreme economic losses to the animal husbandry industry. Once inside cells, *Brucella* resides in a vacuole, designated the BCV, which interacts with components of the endocytic and secretory pathways to ensure bacterial survival. Numerous studies conducted recently have revealed that *Brucella*’s ability to cause a chronic infection depends on how it interacts with the host. This paper describes the immune system, apoptosis, and metabolic control of host cells as part of the mechanism of *Brucella* survival in host cells. *Brucella* contributes to both the body’s non-specific and specific immunity during chronic infection, and it can aid in its survival by causing the body’s immune system to become suppressed. In addition, *Brucella* regulates apoptosis to avoid being detected by the host immune system. The BvrR/BvrS, VjbR, BlxR, and BPE123 proteins enable *Brucella* to fine-tune its metabolism while also ensuring its survival and replication and improving its ability to adapt to the intracellular environment.

## 1 Introduction

Brucellosis is a worldwide zoonotic disease that has brought great harm to both biosecurity and economic development. Some species of the genus *Brucella* can cause brucellosis, whose virulence is mainly reflected in its entry into cells and in its ability to survive and replicate (Gorvel and Moreno, 2002). Erythritol has been considered as an important factor in the pathogenesis of *Brucella abortus* 2308 and its ability to cause abortion in ruminants. Macrophages are immune cells, and their main function is to cause immune reactions and produce immune responses. Macrophage is one of the main target cells of *Brucella* infection. The survival and replication of *Brucella* in macrophages represent one of the strategies for *Brucella* to evade the host’s immune response, and it is also the reason for the failure of some patients to use anti-*Brucella* treatment. In addition, the prolonged existence of *Brucella* in macrophages will affect the signal pathway of the cell, and trigger a complex host response, so that it can adapt to the intracellular environment and reproduce widely in the host cell without destroying the basic cell function (Chaves-Olarte et al., 2002). Compared with acute diseases, the pathogens of chronic diseases have more virulence genes to ensure the persistence of infection (Hong et al., 2000). The intracellular environment allows *Brucella* to coordinate gene expression during infection. The host body specific internal resistance induced expression of genes is usually also an important virulence factor (Boschiroli et al., 2001).

## 2 Chronic intracellular infection with *Brucella* spp.

*Brucella* is a gram-negative facultatively intracellular bacteria that can invade and persist within the host cells and lead to chronic infections. Domestic animals, wild animals, and humans are susceptible to *Brucella*. There are six classical species: *Brucella abortus (B. abortus), Brucella melitensis (B. melitensis), Brucella suis (B. suis), Brucella canis (B. canis), Brucella ovis (B. ovis) and Brucella neotomae (B. neotomae)* (Erkyihun et al., 2022; Kurmanov et al., 2022). Among them, *B. suis*, *B. abortus*, and *B. melitensis* are the most harmful to humans (Olsen and Palmer, 2014). *Brucella* expresses atypical virulence factors, including lipopolysaccharide (LPS), virulence regulatory proteins, and phosphatidylcholine, but lacks classical virulence factors, such as invasive proteases, toxins, or virulence plasmids (Roop et al., 2003). *Brucella* is classified into two types based on differences in the structure of pathogenic bacteria LPS: smooth *Brucella* and rough *Brucella* (Stranahan and Arenas-Gamboa, 2021). Smooth LPS (S-LPS) consists of a polysaccharide O-chain, core, and lipid A, whereas rough strains lacked the O-side chain. The importance of the O-chain for the virulence of naturally occurring smooth *Brucella* strains is well documented. In general, rough (R) type *Brucella* shows reduced virulence, except for *B. ovis* and *B. canis* (Lopez-Santiago et al., 2019).

Brucellosis is typically divided into three distinct phases: the incubation phase before clinical symptoms are evident (within 2 days after infection), the acute phase during which time the pathogen invades and disseminates in host tissue (within 2 days to 3 weeks after infection), and the chronic phase that can eventually result in severe organ damage and death of the host organism (6 months to 1 year or more). (Grillo et al., 2012). It is generally believed that innate immunity is not highly activated during the incubation period. This allows *Brucella* to spread throughout the reticuloendothelial system and establish a replication mechanism within the phagocytes. There are no typical endotoxin symptoms (mainly the lipid A of LPS) during this process, which is different from other gram-negative infections. This clinical observation is consistent with brucellosis (Martirosyan et al., 2011). In the early stage of *Brucella* infection, *Brucella* barely activates the complement system and induces minimal levels of cytokines, the recruitment of pro-inflammatory cells at the infected site is poor (Barquero-Calvo et al., 2007). Since *Brucella* hardly activates the complement system through the classical pathway or activates granulocytes, it causes very little tissue damage and does not cause obvious blood changes, such as leukocytosis, increased neutrophilia, and decreased platelets, coagulation lesions rarely occur during the incubation period (Barquero-Calvo et al., 2007). After the incubation period, strong adaptive immunity begins to appear, and obvious clinical symptoms are observed, such as abortion and infertility in animals and high, undulating fever in humans. In the acute phase, *Brucella* begins to replicate actively in macrophages and dendritic cells (DC). One of the distinguishing features of *Brucella* is that the levels of chemokines and cytokines produced by infected macrophages and DC are low and prolonged, the production of proinflammatory cytokines released by polymorphonuclear (PMN) cells is low, and the activation and demand of natural killer cells (NK) are low (Barquero-Calvo et al., 2007). Although nonspecific immunity is used to control the proliferation of *Brucella* in the acute phase in mice, effective specific immunity is required in the later stage (Grillo et al., 2012).

The first line of defense against *Brucella* includes the phagocytosis action of PMN, macrophages, dendritic cells, NK cells, chemokines, pattern recognition receptors (PRR), and the complement system (Diacovich and Gorvel, 2010; Jiao et al., 2021). The specific immune response caused by *Brucella* infection has three main mechanisms. The first is the secretion of interferon by CD4^+^ T cell, CD8^+^ T cell, γδ T cell, which activates the bactericidal function of macrophages and prevents *Brucella* intracellular survival; the second is the cytotoxic effect of CD8^+^ T cells, which can kill infected macrophages; and the third is Th1 antibody subtypes, such as IgG2a and IgG3, which promote phagocytosis (Martirosyan et al., 2011; Grillo et al., 2012). Furthermore, cytokines such as interleukin-12 (IL-12), interferon-γ (IFN-γ), and tumor necrosis factor (TNF) are important in initiating both specific and non-specific immune responses. *Brucella* can participate in the regulation of innate immune mechanisms and the maintenance of intracellular replication by inhibiting Toll-like receptors (TLR) signaling pathways, the complement system, phagocytes, and apoptosis. Several immunomodulatory molecules, for example, proline racemase protein A (prpA) and the TIR domain-containing protein (TcpB) can influence the Th1 immune response by inhibiting the secretion of IFN-γ and promoting the secretion of interleukin-10 (IL-10) (Alaidarous et al., 2014; Spera et al., 2014). Due to an increase in CD4^+^ and CD25^+^ T cells in the spleen, chronic *Brucella* infection causes immunosuppression in the body (Bahador et al., 2014). The reduced recruitment of macrophages and DCs after *Brucella* infection leads to a decrease in CD8^+^ T lymphocyte activation, thus forming immunosuppression, which is conducive to immunosuppression being beneficial to the replication and chronic infection of *Brucella* (Pasquali et al., 2010).

In the initial stage of infection, macrophages, trophoblastic cell (TE), and other phagocytic cells are the main targets of *Brucella* infection (Xiao et al., 2022). For *Brucella*, they are not only the sites for both survival and replication but also a vehicle for transmission to other organ systems. Macrophages play a key role in the clearance and control of intracellular pathogens. Macrophages can not only kill pathogens and carry out non-specific immunity but also participate in antigen uptake and processing steps to initiate specific immunity. Meanwhile, its secreted cytokines, such as IL-1, IL-6, and IFN-γ, regulate the immune response and activate more lymphocytes and macrophages, playing a role in regulating the immune response and inflammatory immunity (Weiss and Schaible, 2015). Macrophages can eliminate most of the *Brucella* invading the body. However, there is still a small part of *Brucella* that can evade the immune system and use macrophages, as the host to invade, survive, and reproduce.

In its long evolutionary history, *Brucella* has interfered with both specific and nonspecific immune responses to establish a persistent infection, making it difficult to remove thoroughly. However, the specific and comprehensive mechanism of *Brucella* intracellular survival remains unknown.

## 3 *Brucella* mediates autophagy

More and more evidence suggest that nonspecific host immunity is important for *Brucella* intracellular infection. Autophagy is a non-specific immune process based on lysosomes, which can decompose non-essential cells or invading pathogens into cellular components to promote cell survival and provide more energy sources for cells. Some pathogens have evolved strategies to in turn use autophagy to survive inside cells, and *Brucella* can use the cell autophagy mechanism for intracellular replication to establish a good living environment. The specific processes include: quickly escaping from the phagocytic corpuscle to enter the cytoplasm; delaying the maturation of the phagocytic corpuscle at different stages before fusion with lysosomes; surviving and replicating in the degraded phagocytic environment; and completely or partially avoiding the endocytic pathway. All of these processes require microbiological agents to interfere with the function of macrophages (Celli, 2006).

First, *Brucella* relies on specific lipid rafts to enter macrophages. The outer membrane of *Brucella* consists of phospholipids, guanylic acid, lipoproteins, and nonstandard LPS, which replace the long aliphatic hydrocarbon chains. The content of negatively charged sugars in *Brucella* lipid A and core oligosaccharides (BR-LPS) is low; moreover, O-chains and associated polysaccharides are composed of homopolymers of non-reducing N-formyl peroxide sugars. These features help to reduce the negative charge on the surface of the bacteria. This special cell membrane structure prevents *Brucella* from combining with complement (both the classical and the MBL-mediated pathways), bactericidal defensin, bacitracin, or any other cationic bactericidal molecule and is effective against most bactericidal substances in lysosomal extracts, lysozyme, phospholipase, and lactoferrin(Fernandez-Prada et al., 2001; Cardoso et al., 2006). Specifically, the LPS O-chain modifies the fusion properties of BCV membranes or interacts with specific receptors located in lipid rafts to determine the entry of permissive cells. The type A scavenger receptor (SR-A) is considered as the receptor that binds to *Brucella* LPS, respectively (Watarai et al., 2003; Kim et al., 2004). The LPS O-chain also promotes *Brucella* survival by delaying fusion with the lysosome. This process is further enhanced by the disruptive effect of cyclic β-1,2-glucan on BCV lipid rafts (Celli, 2006). Cyclic β-1,2-glucan is secreted by intracellular Brucella, which disrupts cholesterol-rich lipid rafts located on the membrane of BCV and interferes with BCV maturation, thus preventing lysosomal fusion. A deletion mutant of Cgs, the gene encoding the synthetase of cyclic β-1,2-glucan, is unable to avoid fusion with the lysosome, suggesting that the production of cyclic β-1,2-glucan is necessary for *Brucella* intracellular cycle (Bohin, 2000; Arellano-Reynoso et al., 2005).

**Figure 1:**
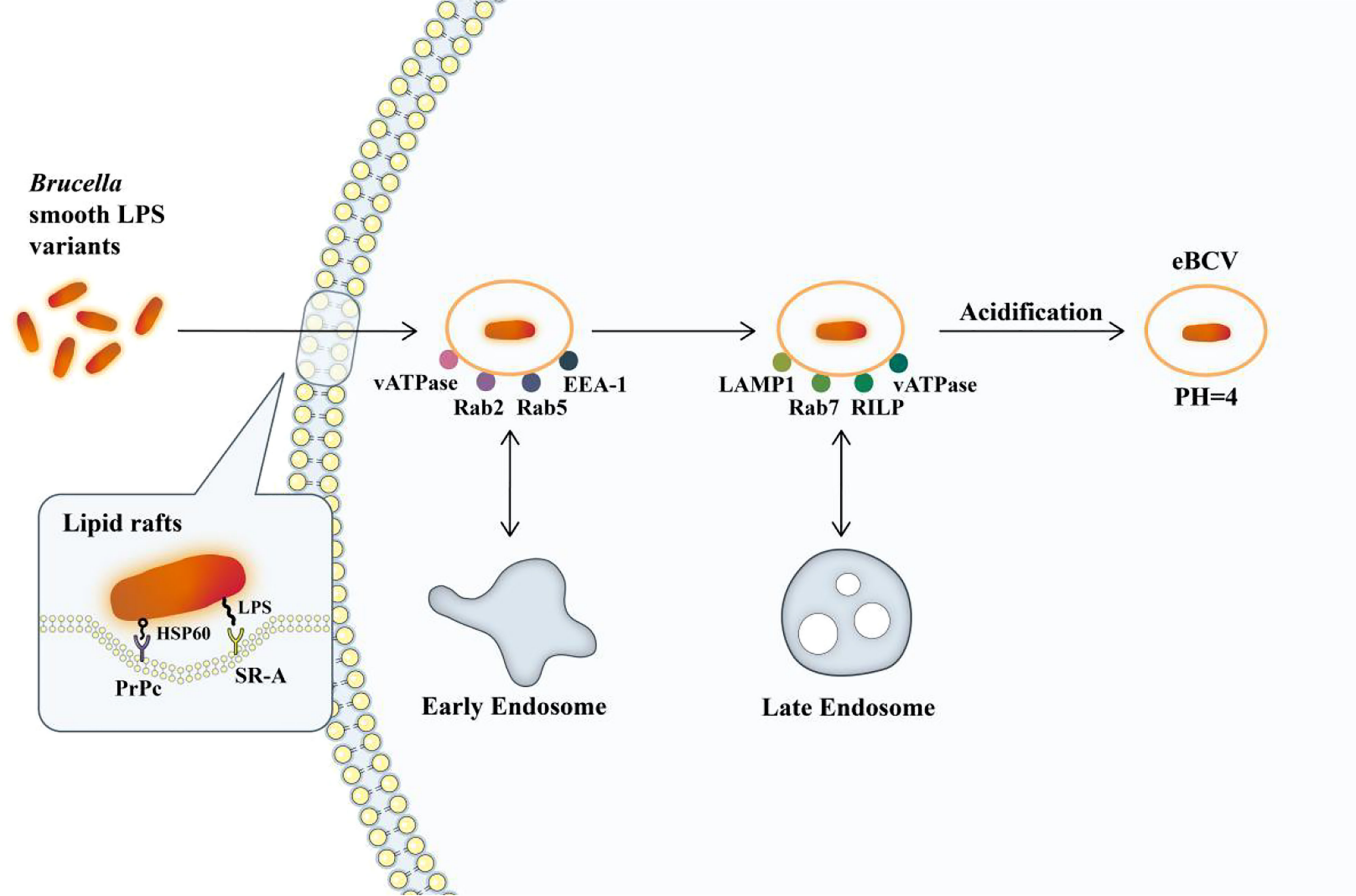
The formation of eBCV. *Brucella* enters macrophages *via* lipid rafts. Hsp60 and *Brucella* LPS bind to PRPC and SR-A receptors on lipid rafts. *Brucella* enters the cell and remains in the membrane envelope cavity, forming BCV containing *Brucella*. It interacts with early endosomes to obtain small GTPase Rab5 and early endosomal antigen (EEA-1) and subsequently obtains markers of late endosomes, such as membrane proteins recombinant lysosomal associated membrane protein 1 (LAMP1) and small GTPase Rab7. It will then be acidified, and the pH will reach 4, which is essential for the survival of *Brucella* and for the intracellular expression of the VirB T4SS (Porte et al., 1999; Boschiroli et al., 2002). The single arrows represent the flow of Brucella intracellular processes, and the double arrows represent interactions.

Upon entry into host cells, the *Brucella* reside in acidified phagosomal compartments known as endosomal *Brucella*-containing vacuoles (eBCVs). The eBCV stage is a necessary step in the intracellular circulation of *Brucella*. With the early and late interaction within the stage, most of the contents of BCV are subjected to enzymatic degradation, and 90% of internal *Brucella* are hydrolyzed and killed. However, the remaining 10% escaped from the host’s killing mechanisms through unknown mechanisms (Ke et al., 2015). This entire maturation process is in line with the complete maturation process, but after maturation, BCV still avoids fusion with the terminally degraded lysosomes, thus ensuring the intracellular survival of bacteria. This vacuole in this process is called eBCV (**Figure 1**). The sustained avoidance of macrophage degradation also requires VirB type IV secretion system (T4SS) and the conversion of eBCV into an endoplasmic reticulum-derived replicating compartment (rBCV). eBCV can provide the conditions necessary to induce the expression of the VirB operon that encodes a type IV secretion system (T4SS) and its transformation into rBCV, including lysosomal pH. In addition, eBCV can trigger intracellular bacterial growth before rBCV is formed (Porte et al., 1999; Celli, 2019).

eBCV will gradually lose its endosomal marker and begin to continuously interact with the endoplasmic reticulum (ER) structure after interaction with the endosome. The site of action is the ER export site covered with coated vesicles II (COPII), and the interaction depends on the VirB IV secretion system, which controls its organization and function through COPII, and its activity is controlled by the small GTPase Sar1. The newly formed vesicles and tubules are fused with the endoplasmic reticulum-Golgi intermediate compartment (ERGIC) or VTC, and the coated vesicles I (COPI), controlled by Arf1 GTPase, are transported to the Golgi apparatus or ER to play a role. Vacuoles containing VirB-deficient *Brucella* cannot sustain interactions and fuse with the ER. They eventually fuse with lysosomes. Studies have shown that eBCV interacts with the COPII coating structure of the proven functional endoplasmic reticulum exit sites (ERES) but doesn’t interact with the COPI coating structure (Celli et al., 2005; Xiong et al., 2021). Finally, eBCV obtains markers related to the ER membrane, such as calcium-binding protein, Sec61, and Pdi, indicating the turnover of the eBCV membrane and the accumulation of ER-derived membrane. At the same time, these vacuoles also acquire the structure and functional characteristics of ER, which further indicates that eBCV is gradually derived from ER (Pizarro-Cerda et al., 1998; Comerci et al., 2001; Celli et al., 2003). These changes in structure and function are associated with the initiation of bacterial replication. The vacuoles are then called rBCV, where the bacteria replicate.

The effectors of T4SS, BspA, BspB, and BspF inhibit the secretion of host proteins and promote bacterial replication (Myeni et al., 2013). Although it is not yet clear how BspA and BspF work, how BspB works has been revealed. The production of rBCV and the replication of bacteria are inseparable from BspB. BspB is delivered to the Golgi apparatus of host cells and interacts with the conserved oligomeric Golgi (COG) complex. As a result, the function of COG is changed, and the reverse Golgi vesicles that depend on COG are transported to BCV, thereby obtaining the Golgi apparatus source membrane. It is also known that the T4SS effector, RicA, is involved in controlling the formation of rBCV (Miller et al., 2017). At the same time, the unfolded protein response (UPR) transmembrane sensor, inositol-requiring enzyme-1 (IRE1), is also necessary for bacterial replication (Pandey et al., 2018). YPT-interacting protein 1A (Yip1A) is produced when IRE1 is activated, and it is then phosphorylated when combined with IRE1, which triggers XBP-1-dependent transcription (Taguchi et al., 2015). This activation also acts on the upregulation of COPII-coat complex subunits (Taguchi et al., 2015). The COPII-coat complex is essential for the formation of rBCV and is a crucial part of ERES and early secretory transport (Celli et al., 2005).

**Figure 2:**
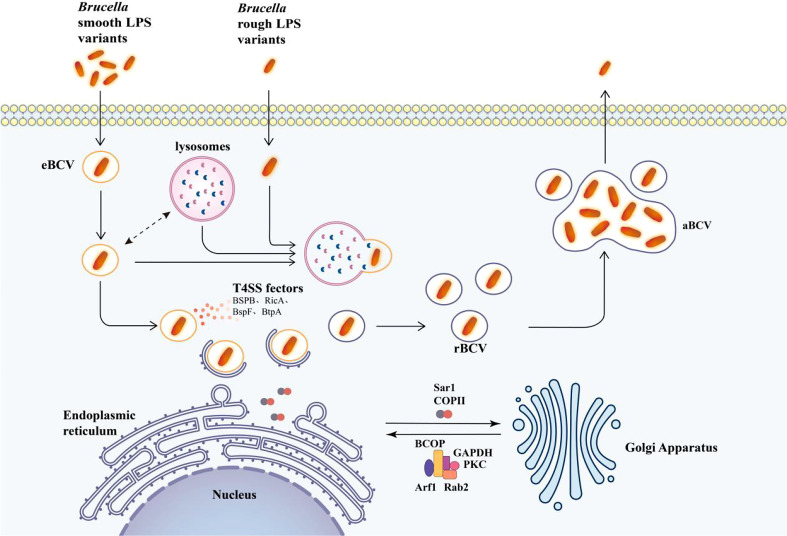
Model of intracellular transport of *Brucella* in macrophages. The lysosome interacts with eBCV and activates the transcription of T4SS effector proteins. T4SS effectors facilitate the arrival of eBCV at the endoplasmic reticulum exit site, and eBCV interacts with the surface coating of ERES to obtain the structural and functional characteristics of ER to form rBCV. The autophagy initiation proteins ULK1, ATG14L, and Beclin-1 play an important role in the formation of aBCV and finally release the pathogen from the cell. The mechanism of entry of *Brucella* rough LPS variants into cells is not clear, and after entering the cell, *Brucella* is degraded by lysosomal phagocytosis upon entry. The single arrows represent the flow of Brucella intracellular processes, and the double arrows represent interactions.

After extensive bacterial replication, autophagy BCV (aBCV) is produced (**Figure 2**). The production of aBCV requires typical autophagy nucleation but does not require the extended complex. Therefore, the lack of Beclin-1, ULK1, and Atg14 will prevent its formation, but the lack of Atg5, Atg7, Atg4, or Atg16L will not affect it. This is also the performance of *Brucella* using autophagy to complete its intracellular circulation. aBCV has the characteristics of advanced ribosomes, which are consistent with the mature autophagosome without ER markers, so its function is different from that of rBCV, but it is closely related to the release of bacteria and the spread between cells (**Figure 2**) (Boschiroli et al., 2002). The bacteria are released from the pores of the cell membrane into the intercellular substance, causing cell lysis. During the cell-to-cell propagation of *Brucella*, smooth (S) type *Brucella* may dissociate and become an R type. This dissociation is also enhanced in an acidic environment, facilitating the spread of *Brucella* from acidic phagocytosis. It is important to note that after dissociating into R type *Brucella*, S type *Brucella* can revert to S type. Therefore, S type *Brucella* may become R type when it needs to spread and then revert to S type when it escapes to resist intracellular killing. Then, *Brucella* will infect more macrophages and begin a new cycle of replication and dissociation. Rough mutants may be killed by complement or another cationic peptide-mediated cleavage (Pei et al., 2014).

## 4 *Brucella* regulates metabolism

**Figure 3:**
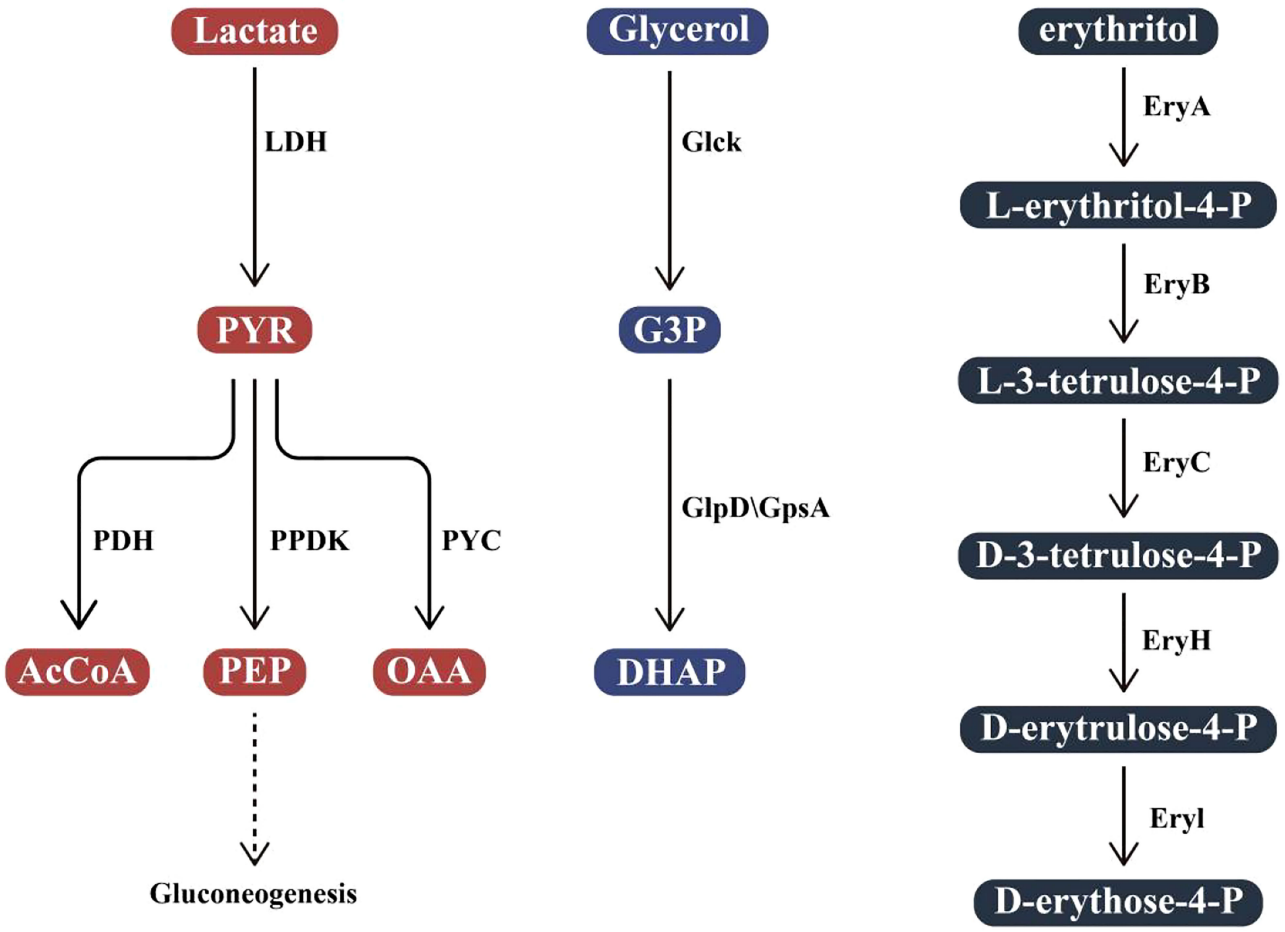
Utilization of polyols In *Brucella.* Lactate is converted into pyruvate (PYR) by L-lactate dehydrogenase (LDH). PYR is then phosphorylated by pyruvate phosphodikinase (PPDK) to produce phosphoenolpyruvate (PEP) and enter the gluconeogenesis pathway, converted to acetyl coenzyme A (AcCoA) by pyruvate dehydrogenase (PDH) or to oxaloacetate (OAA) by pyruvate carboxylase (PYC); Glycerol is converted to glyceraldehyde 3-phosphate (G3P) by glycerol kinase (GlcK). G3P is further activated by G3P dehydrogenase (GlpD) or G3P dehydrogenase (GpsA) to produce 3,4-dihydroxy acetophenone (DHAP). In B abortus, erythritol is first phosphorylated by EryA to L-erythritol-4-P, then L-erythritol-4-P is oxidized by EryB to L-3-tetramethysaccharide-4-P and then transformed into D-erythrocyte 4-P, catalyzed by EryC, EryH, and EryI in turn.

The ability to regulate its metabolism is also one of the keys to the successful adaptation of *Brucella in vivo.* Metabolic systems adapted to intracellular survival can make better use of nutrients at all stages of the infectious cycle (Brown et al., 2008; Lamontagne et al., 2009). When *Brucella* enters a cell and persists, its metabolic system adapts. The regulation of its metabolic system enables *Brucella* to take advantage of the metabolic pathways and intermediates provided by the host to adapt to various environmental conditions in the host cell. To adapt to different environments at different stages of the infection cycle, a comprehensive fine-tuning of gene expression is required to alter the corresponding functions of the bacteria. *B. abortus* is considered to have a slow metabolism during the period from entering the cell to starting to replicate (De Bolle et al., 2015). The protein obtained through sugar absorption, pentose phosphate pathway (PP), and tricarboxylic acid cycle (TCA) pathways, as well as the biosynthesis of amino acids, purines, and pyrimidines, is reduced. *Brucella*, on the other hand, does not store glycogen or poly-β-hydroxybutyrate, so it can maintain basic metabolism *via* protein and amino acid catabolism (Chain et al., 2005). It has also been suggested that *B. abortus* can even use macromolecules such as ribosomes at this stage. After entering the replication niche, metabolism began to strengthen (Lamontagne et al., 2009). Different strains of *Brucella* have different carbon sources. *B. suis* biovars 1 and 5, *B. microti*, and *B. neotomae* use C5 sugars such as xylose, arabinose, and ribose as the sole carbon source; some strains of *B. abortus*, *B. melitensis*, and *B. suis* use galactose as the sole carbon source when tested on the vitamin and mineral based medium; at the same time, some *B. melitensis* and *B. suis* strains grew on fructose and mannose as carbon sources (McCullough and Beal, 1951). *Brucella* breaks down hexose through the PP pathway and the incomplete Embden-Meyerhof-Parnas glycolytic pathway (EMP), and then further metabolites through TCA. But for the three most representative species of *Brucella*: *B. abortus*, *B. melitensis*, and *B. suis*, hexose is not the carbon source of choice. Instead, they preferentially utilize a four-carbon sugar alcohol (erythritol) and catabolize it to produce a trisaccharide phosphate. In addition, other studies have shown that some strains of *Brucella* could use polyols such as lactic acid and glycerol as peripheral carbon sources (**Figure 3**) (Barbier et al., 2018). The metabolic pathways of *Brucella* mainly include active PP and TCA cycles, potentially active Entner-Doudoroff (ED) and glyoxylate pathways, and incomplete EMP. Among them, the PP pathway plays an important role in the production of biological precursors and the degradation of sugars (Barbier et al., 2011).

The two-component regulation system of *Brucella*, BvrR/BvrS, is the most distinctive two-component sensory regulation system of *Brucella* so far. BvrS is a membrane-bound homologous dimer protein belonging to the histidine protein kinase superfamily. It has three conserved regions: an amino-terminal periplasmic sensing domain with transmembrane segments, a cytoplasmic dimerization domain with a specific His residue, and the carboxy-terminal ATP-binding kinase domain. BvrR is a cytoplasmic protein that is highly similar to the reaction-regulating protein OmpR/PhoB subfamily, and its specific Asp residues are located in the conserved regulatory domain and have the effect domain of DNA binding activity (Lopez-Goni et al., 2002). It was originally discovered that BvrR/BvrS regulated the homeostasis and structure of several proteins in *B. abortus* cell membranes. However, with further research, it has been found that it also has functions related to metabolic function, thus contributing to the adaptation of *B. abortus* to an intracellular lifestyle (Lamontagne et al., 2009). Upon exposure to specific environmental stimuli, BvrS autophosphorylated on conserved histidine residues and mediated phosphate transfer to conserved aspartic acid on BvrR. The latter regulates cell expression through the differential expression of target genes. BvrS/BvrR is important for the virulence of bacteria, and translocation inactivation leads to defects in attachment, invasion, and intracellular replication (Sola-Landa et al., 1998). A recent transcriptional analysis shows that the BvrR mutation had significant effects on the expression of genes associated with carbohydrate, amino acid, fatty acid, and nitrogen metabolism (Viadas et al., 2010).

Quorum sensing (QS) is a regulatory system that can regulate gene expression at the population level according to the density of local bacteria. Recent transcriptional and proteomic analyses have shown that the inactivation of the two QS regulators, VjbR and BabR, has a strong effect on the genes involved in metabolism, especially the genes encoding the TCA cycle and glycolysis (Uzureau et al., 2010; Weeks et al., 2010). Since *B. abortus*’s intracellular expansion is limited until the replication site is reached, in response, *B. abortus* uses VjbR to slow down the metabolism of *Brucella* until it reaches endoplasmic reticulum derived rBCV. Subsequently, the BabR regulator acts on the reactivation of basal metabolism. BvrR can also activate the transcription of VjbR, so the two regulatory systems seem to be related (Martinez-Nunez et al., 2010; Viadas et al., 2010). To sum up, BvrS/BvrR, TCS, and QS systems are helpful to the regulation of *B. abortus* metabolism in the intracellular inventory.

The phosphoenolated pyruvate phosphotransferase system (PTS) provides an integrated system for bacteria to ensure optimal utilization of carbohydrates in complex environments, a feature that also plays an important role in host-bacterial interactions. The three genes, *hprK*, *ptsM*, and *ptsO*, are found downstream of the conserved two-component system genes (*BvrS*/*BvrR*, *Exos*/*ChvI*) associated with infection or symbiosis in all pathogenic or symbiotic -proteobacteria (Sola-Landa et al., 1998; Belanger et al., 2009). This genome structure shows a functional link between PTS and BvrS/BvrR (Boel et al., 2003; Barabote and Saier, 2005). Both *BvrR* and *ptsP* seem to modulate the expression of the QS modifier VjbR. VjbR, in turn, regulates virulence and metabolic determinants.

BPE123 is a T4SS effector protein of *Brucella*, indicating that the bacteria may manipulate the host carbohydrates synthesis or decomposition pathways through T4SS effector molecules. BPE123 interacts with the key glycolysis/gluconeogenesis host enzyme α-enolase, allowing it to bind to BCV and induce structural or functional changes that result in α-enolase activation. When this enzyme is depleted by RNA interference, the intracellular replication of *B. abortus* in HeLa cells is impaired, thus confirming the role of this protein in the infection process (Marchesini et al., 2016). Current data indicates that T4SS and its effector proteins could regulate the metabolic pathways of host cells and contribute to the intracellular survival of bacteria. However, how to control the secretion and action of effector proteins is still unclear (Hayek et al., 2019).

## 5 *Brucella* regulates apoptosis

Regulating the macrophages’ apoptosis is also one of the strategies used by *Brucella* to achieve intracellular persistence. By regulating the apoptosis of these host cells, especially macrophages, *Brucella* can reduce the bactericidal ability of immune cells. *Brucella* can promote or inhibit apoptosis in different conditions. Studies have shown that S type *Brucella* inhibited macrophage apoptosis, while attenuated R type *Brucella* induced macrophage apoptosis (Im et al., 2016). This may be related to the dissociation of the bacteria when they are released from the target cell. Gross et al. demonstrated that *B. suis* could disrupt the TNF-α apoptosis pathway by triggering cell signal transduction, by blocking the core steps of cell apoptosis (Gross et al., 2000). Galdeiro et al. found that the apoptosis of cells challenged with the *B. abortus* S19 strain was delayed when compared with lymphocytes and monocytes from healthy controls (Galdiero et al., 2000). These results suggest that Brucella is involved in the induction apoptosis, indicating that the immune system of the host in turn adapts them to infection.

Brucella infection induced the expression of zinc finger protein A20 in macrophages. A20, also known as tumor necrosis factor alpha-induced protein 3 (TNFAIP 3), is a dual inhibitor of macrophage activation and apoptosis, A20 has important physiological functions (Priem et al., 2020). On the one hand, activation of nuclear factor-κB (NF-κB) can inhibit apoptosis induced by tumor necrosis factor receptor 1 (TNFR1), and A20 can terminate the activity of NF-κB, so A20 can promote cell apoptosis (Vallabhapurapu and Karin, 2009; Vereecke et al., 2009). *B. melitensis* infection, on the other hand, significantly increases the expression of the TNF-α gene in macrophages, and A20, as one of the genes induced by TNF-α, also increases significantly (Wang et al., 2011). TNF-α induces macrophage apoptosis by signaling through complex I, Tradd-TRAF2-RIP (Micheau and Tschopp, 2003). A20 can ubiquitinate these proteins for degradation (He and Ting, 2002; Wertz et al., 2004). Therefore, A20 also has an anti-apoptotic function in macrophages. The results of Wei et al. revealed that A20 is involved in the inhibition of macrophage apoptosis in the process of *B. abortus* infection. The lack of A20 will inhibit the growth of *B. abortus* in macrophages, but it is not enough to trigger the apoptosis of macrophages. *B. abortus* induces A20 to promote *B. abortus* intracellular growth by inhibiting macrophage activation and apoptosis. This study provides a new explanation for the ability of *B. abortus* to grow and replicate in macrophages in the early stages of infection (Wei et al., 2015).

Reactive oxygen species (ROS) are the second messenger of apoptosis (Carrasco et al., 2016). When cells receive apoptosis signals, ROS levels increase, which may lead to increased Ca^2+^ influx, upregulation of Bax, the opening of the mitochondrial permeability transition pore (MPTP), activation of trypsin, and eventual cell death (Sun et al., 2016). Different levels of ROS determine apoptosis, necrosis, or the transformation from apoptosis to necrosis. Excessive ROS can change the activity of specific enzymes through redox reactions and participate in the regulation of autophagy and programmed cell death, thus adversely affecting the body. Therefore, ROS increased after apoptosis, which in turn promoted apoptosis. The anti-apoptotic protein BCL-2 inhibits ROS-induced lipid peroxidation by inhibiting ROS production. *B. melitensis* 16M can regulate the effects of the AIR domain on inflammatory factors, autophagy, and apoptosis in mouse macrophages through the ROS signaling pathway. The ability of *B. melitensis* 16M to promote apoptosis increased with infection time. AIR can also influence *B. melitensis* 16M-induced apoptosis *via* the ROS pathway (Li et al., 2016).

Calcium-activated cysteine protease 2 (Calpain-2) regulates macrophage apoptosis and necrosis under a variety of pathological conditions. Normally, an increase in intracellular calcium leads to the activation of Calpain-2, which induces macrophage apoptosis. On the other hand, *B. abortus* infection inhibits macrophage apoptosis by increasing intracellular calcium content. Nedd4 participates in apoptosis by ubiquitination and degradation of its substrates such as PTEN and caspase-9 (Ahn et al., 2008; Fombonne et al., 2012). Caspase-3 is one of the most important executors of apoptosis, and it plays a major role in the process of apoptosis (Cryns and Yuan, 1998). Current studies have shown that *B. abortus* infection triggers the degradation of Calpain-2 by activating Nedd4, and prevents the activation of the apoptotic effector caspase-3, thereby, inhibiting macrophage apoptosis. Calpain-2 is ubiquitinated by Nedd4, after infection with *B. abortus*, and degraded as intracellular calcium increases. These results indicate that after infection, *B. abortus* inhibits macrophage apoptosis through Nedd4-dependent Calpain-2 degradation (Cui et al., 2014).

JAK2/STAT3 signal transduction pathway is an important intracellular signal transduction pathway, as well as a common pathway for many cytokines and growth factors. It plays an essential role in cell proliferation, differentiation, apoptosis, and immune regulation. *B. melitensis* M5-90 infection regulates the apoptosis and proinflammatory response of RAW264.7 cells by activating the JAK2/STAT3 signaling pathway. AG490 is an inhibitor that inhibits JAK2 activity in macrophages of mice infected with *B. melitensis* M5-90 (Luo and Laaja, 2004). AG490 at various concentrations modulated the activation of the JAK2/STAT3 pathway to varying degrees but essentially inhibited TNF-α expression. TNF-α is involved in the induction of several distinct immune responses to intracellular infections (Aggarwal, 2003). Furthermore, TNF-α-mediated apoptosis is involved in the pathology of chronic inflammation and autoimmune diseases (Wang et al., 2009). TNF-α activates TNF-α receptor-1 and induces apoptosis by activating caspase in the death receptor pathway (Ashkenazi, 2002), implying that TNF-α can trigger macrophage apoptosis via the JAK2/STAT3 pathway. At the same time, TNF-α can also activate caspase-8 and caspase-3 to cause apoptosis (Dbaibo et al., 1997), and BCL-2 can regulate this effect. BCL-2 is an anti-apoptotic gene. Bax is a member of the BCL-2 family and can promote apoptosis (Karabay et al., 2014). The results show that the expression of caspase-3 and Bax decreased in RAW264.7 cells infected with *B. melitensis* M5-90 treated by AG490, whereas the expression of BCL-2 shows the opposite effect. In summary, *B. melitensis* M5-90 activates the JAK2/STAT3 signaling pathway and regulates TNF-α-induced apoptosis. Inhibition of the JAK2/STAT3 pathway can inhibit the Th1 immune response, inhibit apoptosis, and contribute to the intracellular survival of *B. abortus* (Yi et al., 2018).

Other studies have shown that BR-LPS O chain polysaccharide is also involved in the prevention of macrophage apoptosis. Zhang et al. found that *Brucella* outer membrane protein Omp31 inhibited TNF-α-mediated apoptosis during *Brucella* infection of macrophages (Zhang et al., 2016). The study by Liu also proved that the Omp31 protein could inhibit the apoptosis of microglia (Liu and Ma, 2017).

## 6 Conclusions

The interaction mechanism between pathogen and host is very complex, involving many biological factors and pathways. It is known that the adaptive regulation of immune response, metabolism, and apoptosis caused by *Brucella* after infection is necessary for its intracellular persistence and replication, and some of these biological factors and pathways have multiple roles. Although there have been a lot of related reports, there are still many issues worthy of further study.

## Author contributions

Writing-original draft preparation, XG, HZ, ML, YX, and GG; writing-review and editing, ZS, XS, and JG; visualization, QH; supervision, BZ, and YC; project administration, YC and HJ; funding acquisition, YC and H.J. All authors contributed to the article and approved the submitted version.

## Glossary

**Table:**

AcCoA	Acetyl coenzyme A
*B. abortus*	*Brucella abortus*
BCL-2	B-cell lymphoma-2
BCV	*Brucella*-containing Vacuole
*B. melitensis*	*Brucella melitensis*
*B. microti*	*Brucella microti*
*B. neotomae*	*Brucella neotomae*
*B. ovis*	*Brucella ovis*
BR-LPS	core oligosaccharides
*B. suis*	*Brucella suis*
Calpain-2	Calcium-activated cysteine protease
COG	Conserved oligomeric Golgi
COPI	Coated vesicles I
COPII	Coated vesicles II
DC	Dendritic cells
DHAP	3,4-dihydroxyacetophenone
ED	Entner-doudoroff
EEA-1	Early endosomal antigen
EMP	Embden-meyerhof-parnas glycolytic pathway
ER	Endoplasmic reticulum
ERES	Endoplasmic reticulum exit sites
ERGIC	Endoplasmic reticulum-Golgi intermediate compartment
GlcK	Glycerol kinase
GlpD	G3P dehydrogenase
GpsA	G3P dehydrogenase
G3P	Glyceraldehyde 3-phosphate
Hsp60	Heat shock protein 60
IFN-γ	Interferon-γ
IL-10	Interleukin-10
IRE1	Inositol-requiring enzyme-1
LAMP1	Recombinant lysosomal associated membrane protein 1
LDH	L-lactate dehydrogenase
MPTP	Mitochondrial permeability transition pore
NF-kB	Nuclear factor-kB
NK	Natural killer cell
OAA	Oxaloacetate
PDH	Pyruvate dehydrogenase
PEP	Phosphoenolpyruvate
PMN	Polymorphonuclear
PP	Pentose phosphate pathway
PPDK	Pyruvate phosphodikinase
PrPc	Cellular prion protein
PRR	Pattern recognition receptors
PTS	Phosphoenolated pyruvate phosphotransferase system
PYC	Pyruvate carboxylase
PYR	Pyruvate
QS	Quorum sensing
ROS	Reactive oxygen species
R type	Rough type
SR-A	Type A scavenger receptor
S type	Smooth type
TCA	Tricarboxylic acid cycle
TE	Trophoblastic cell
TLR	Toll-like receptors
TNF	Tumor necrosis factor
TNFR1	Tumor necrosis factor receptor 1
T4SS	Type IV secretion system
UPR	Unfolded protein response
Yip1A	YPT-interacting protein 1A

## References

[B1] Aggarwal B. B. (2003). Signaling pathways of the TNF superfamily: a double-edged sword. Nat. Rev. Immunol. 3, 745. doi: 10.1038/nri1184 12949498

[B2] Ahn Y. Hwang C. Y. Lee S. R. Kwon K. S. Lee C. (2008). The tumour suppressor PTEN mediates a negative regulation of the E3 ubiquitin-protein ligase Nedd4. Biochem. J. 412, 331. doi: 10.1042/BJ20071403 18307411

[B3] Alaidarous M. Ve T. Casey L. W. Valkov E. Ericsson D. J. Ullah M. O.. (2014). Mechanism of bacterial interference with TLR4 signaling by brucella toll/interleukin-1 receptor domain-containing protein TcpB. J. Biol. Chem. 289, 654. doi: 10.1074/jbc.M113.523274 24265315 PMC3887194

[B4] Arellano-Reynoso B. Lapaque N. Salcedo S. Briones G. Ciocchini A. E. Ugalde R.. (2005). Cyclic beta-1,2-glucan is a *Brucella* virulence factor required for intracellular survival. Nat. Immunol. 6, 618. doi: 10.1038/ni1202 15880113

[B5] Ashkenazi A. (2002). Targeting death and decoy receptors of the tumour-necrosis factor superfamily. Nat. Rev. Cancer 2, 420. doi: 10.1038/nrc821 12189384

[B6] Bahador A. Hadjati J. Hassannejad N. Ghazanfari H. Maracy M. Jafari S.. (2014). Frequencies of CD4^+^ T regulatory cells and their CD25(high) and FoxP3(high) subsets augment in peripheral blood of patients with acute and chronic brucellosis. Osong Public Health Res. Perspect. 5, 161. doi: 10.1016/j.phrp.2014.04.008 25180149 PMC4147229

[B7] Barabote R. D. Saier M. H. Jr. (2005). Comparative genomic analyses of the bacterial phosphotransferase system. Microbiol. Mol. Biol. Rev. 69, 608. doi: 10.1128/MMBR.69.4.608-634.2005 16339738 PMC1306802

[B8] Barbier T. Nicolas C. Letesson J. J. (2011). *Brucella* adaptation and survival at the crossroad of metabolism and virulence. FEBS Lett. 585, 2929. doi: 10.1016/j.febslet.2011.08.011 21864534

[B9] Barbier T. Zuniga-Ripa A. Moussa S. Plovier H. Sternon J. F. Lazaro-Anton L.. (2018). *Brucella* central carbon metabolism: an update. Crit. Rev. Microbiol. 44, 182. doi: 10.1080/1040841X.2017.1332002 28604247

[B10] Barquero-Calvo E. Chaves-Olarte E. Weiss D. S. Guzmán-Verri C. Chacón-Díaz C. Rucavado A.. (2007). Brucella abortus Uses a Stealthy Strategy to Avoid Activation of the Innate Immune System during the Onset of Infection. PLoS ONE 2 (7), e631. doi: 10.1371/journal.pone.0000631 17637846 PMC1910614

[B11] Belanger L. Dimmick K. A. Fleming J. S. Charles T. C. (2009). Null mutations in sinorhizobium meliloti exoS and chvI demonstrate the importance of this two-component regulatory system for symbiosis. Mol. Microbiol. 74, 1223. doi: 10.1111/j.1365-2958.2009.06931.x 19843226

[B12] Boel G. Mijakovic I. Maze A. Poncet S. Taha M. K. Larribe M.. (2003). Transcription regulators potentially controlled by HPr kinase/phosphorylase in gram-negative bacteria. J. Mol. Microbiol. Biotechnol. 5, 206. doi: 10.1159/000071072 12867744

[B13] Bohin J. P. (2000). Osmoregulated periplasmic glucans in proteobacteria. FEMS Microbiol. Lett. 186, 11. doi: 10.1111/j.1574-6968.2000.tb09075.x 10779706

[B14] Boschiroli M. L. Foulongne V. O'Callaghan D. (2001). Brucellosis: a worldwide zoonosis. Curr. Opin. Microbiol. 4, 58. doi: 10.1016/s1369-5274(00)00165-x 11173035

[B15] Boschiroli M. L. Ouahrani-Bettache S. Foulongne V. Michaux-Charachon S. Bourg G. Allardet-Servent A.. (2002). The *Brucella* suis virB operon is induced intracellularly in macrophages. Proc. Natl. Acad. Sci. U.S.A. 99, 1544. doi: 10.1073/pnas.032514299 11830669 PMC122227

[B16] Brown S. A. Palmer K. L. Whiteley M. (2008). Revisiting the host as a growth medium. Nat. Rev. Microbiol. 6, 657. doi: 10.1038/nrmicro1955 18679171 PMC3115587

[B17] Cardoso P. G. Macedo G. C. Azevedo V. Oliveira S. C. (2006). *Brucella* spp noncanonical LPS: structure, biosynthesis, and interaction with host immune system. Microb. Cell Fact 5, 13. doi: 10.1186/1475-2859-5-13 16556309 PMC1435926

[B18] Carrasco E. Blazquez-Castro A. Calvo M. I. Juarranz A. Espada J. (2016). Switching on a transient endogenous ROS production in mammalian cells and tissues. Methods 109, 180. doi: 10.1016/j.ymeth.2016.08.013 27586523

[B19] Celli J. (2006). Surviving inside a macrophage: the many ways of *Brucella* . Res. Microbiol. 157, 93. doi: 10.1016/j.resmic.2005.10.002 16364608

[B20] Celli J. (2019). The intracellular life cycle of brucella spp. Microbiol. Spectr. 7, 10.1128. doi: 10.1128/microbiolspec.BAI-0006-2019 PMC6448592 30848234

[B21] Celli J. de Chastellier C. Franchini D. M. Pizarro-Cerda J. Moreno E. Gorvel J. P. (2003). *Brucella* evades macrophage killing *via* VirB-dependent sustained interactions with the endoplasmic reticulum. J. Exp. Med. 198, 545. doi: 10.1084/jem.20030088 12925673 PMC2194179

[B22] Celli J. Salcedo S. P. Gorvel J. P. (2005). *Brucella* coopts the small GTPase Sar1 for intracellular replication. Proc. Natl. Acad. Sci. U.S.A. 102, 1673. doi: 10.1073/pnas.0406873102 15632218 PMC547823

[B23] Chain P. S. Comerci D. J. Tolmasky M. E. Larimer F. W. Malfatti S. A. Vergez L. M.. (2005). Whole-genome analyses of speciation events in pathogenic *Brucella* . Infect. Immun. 73, 8353. doi: 10.1128/IAI.73.12.8353-8361.2005 16299333 PMC1307078

[B24] Chaves-Olarte E. Guzman-Verri C. Meresse S. Desjardins M. Pizarro-Cerda J. Badilla J.. (2002). Activation of rho and rab GTPases dissociates *Brucella* abortus internalization from intracellular trafficking. Cell Microbiol. 4, 663. doi: 10.1046/j.1462-5822.2002.00221.x 12366403

[B25] Comerci D. J. Martinez-Lorenzo M. J. Sieira R. Gorvel J. P. Ugalde R. A. (2001). Essential role of the VirB machinery in the maturation of the *Brucella* abortus-containing vacuole. Cell Microbiol. 3, 159. doi: 10.1046/j.1462-5822.2001.00102.x 11260139

[B26] Cryns V. Yuan J. (1998). Proteases to die for. Genes Dev. 12, 1551. doi: 10.1101/gad.12.11.1551 9620844

[B27] Cui G. Wei P. Zhao Y. Guan Z. Yang L. Sun W.. (2014). *Brucella* infection inhibits macrophages apoptosis *via* Nedd4-dependent degradation of calpain-2. Vet. Microbiol. 174, 195. doi: 10.1016/j.vetmic.2014.08.033 25258171

[B28] Dbaibo G. S. Perry D. K. Gamard C. J. Platt R. Poirier G. G. Obeid L. M.. (1997). Cytokine response modifier a (CrmA) inhibits ceramide formation in response to tumor necrosis factor (TNF)-alpha: CrmA and bcl-2 target distinct components in the apoptotic pathway. J. Exp. Med. 185, 481. doi: 10.1084/jem.185.3.481 9053448 PMC2196031

[B29] De Bolle X. Crosson S. Matroule J. Y. Letesson J. J. (2015). *Brucella* abortus cell cycle and infection are coordinated. Trends Microbiol. 23, 812. doi: 10.1016/j.tim.2015.09.007 26497941 PMC8800490

[B30] Diacovich L. Gorvel J. P. (2010). Bacterial manipulation of innate immunity to promote infection. Nat. Rev. Microbiol. 8, 117. doi: 10.1038/nrmicro2295 20075926

[B31] Erkyihun G. A. Gari F. R. Kassa G. M. (2022). Bovine brucellosis and its public health significance in Ethiopia. Zoonoses 2 (1), 15. doi: 10.15212/ZOONOSES-2022-0005

[B32] Fernandez-Prada C. M. Nikolich M. Vemulapalli R. Sriranganathan N. Boyle S. M. Schurig G. G.. (2001). Deletion of wboA enhances activation of the lectin pathway of complement in *Brucella* abortus and *Brucella* melitensis. Infect. Immun. 69, 4407. doi: 10.1128/IAI.69.7.4407-4416.2001 11401980 PMC98513

[B33] Fombonne J. Bissey P. A. Guix C. Sadoul R. Thibert C. Mehlen P. (2012). Patched dependence receptor triggers apoptosis through ubiquitination of caspase-9. Proc. Natl. Acad. Sci. U.S.A. 109, 10510. doi: 10.1073/pnas.1200094109 22679284 PMC3387056

[B34] Galdiero E. Romano Carratelli C. Vitiello M. Nuzzo I. Del Vecchio E. Bentivoglio C.. (2000). HSP and apoptosis in leukocytes from infected or vaccinated animals by *Brucella* abortus. New Microbiol. 23, 271. 10939042

[B35] Gorvel J. P. Moreno E. (2002). *Brucella* intracellular life: from invasion to intracellular replication. Vet. Microbiol. 90, 281. doi: 10.1016/s0378-1135(02)00214-6 12414149

[B36] Grillo M. J. Blasco J. M. Gorvel J. P. Moriyon I. Moreno E. (2012). What have we learned from brucellosis in the mouse model? Vet. Res. 43, 29. doi: 10.1186/1297-9716-43-29 22500859 PMC3410789

[B37] Gross A. Terraza A. Ouahrani-Bettache S. Liautard J. P. Dornand J. (2000). *In vitro brucella* suis infection prevents the programmed cell death of human monocytic cells. Infect. Immun. 68, 342. doi: 10.1128/IAI.68.1.342-351.2000 10603407 PMC97140

[B38] Hayek I. Berens C. Luhrmann A. (2019). Modulation of host cell metabolism by T4SS-encoding intracellular pathogens. Curr. Opin. Microbiol. 47, 59. doi: 10.1016/j.mib.2018.11.010 30640035

[B39] He K. L. Ting A. T. (2002). A20 inhibits tumor necrosis factor (TNF) alpha-induced apoptosis by disrupting recruitment of TRADD and RIP to the TNF receptor 1 complex in jurkat T cells. Mol. Cell Biol. 22, 6034. doi: 10.1128/MCB.22.17.6034-6045.2002 12167698 PMC133997

[B40] Hong P. C. Tsolis R. M. Ficht T. A. (2000). Identification of genes required for chronic persistence of *Brucella* abortus in mice. Infect. Immun. 68, 4102. doi: 10.1128/IAI.68.7.4102-4107.2000 10858227 PMC101704

[B41] Im Y. B. Jung M. Shin M. K. Kim S. Yoo H. S. (2016). Expression of cytokine and apoptosis-related genes in bovine peripheral blood mononuclear cells stimulated with *Brucella* abortus recombinant proteins. Vet. Res. 47, 30. doi: 10.1186/s13567-016-0311-7 26864657 PMC4750197

[B42] Jiao H. Zhou Z. Li B. Xiao Y. Li M. Zeng H.. (2021). The mechanism of facultative intracellular parasitism of *Brucella* . Int. J. Mol. Sci. 22, 3673. doi: 10.3390/ijms22073673 33916050 PMC8036852

[B43] Karabay A. Z. Aktan F. Sunguroglu A. Buyukbingol Z. (2014). Methylsulfonylmethane modulates apoptosis of LPS/IFN-gamma-activated RAW 264.7 macrophage-like cells by targeting p53, bax, bcl-2, cytochrome c and PARP proteins. Immunopharmacol Immunotoxicol 36, 379. doi: 10.3109/08923973.2014.956752 25211405

[B44] Ke Y. Wang Y. W.andChen Z. (2015). Type IV secretion system of Brucella spp. and its effectors. Front. Cell Infect. Microbiol. 5. doi: 10.3389/fcimb.2015.00072 PMC4602199 26528442

[B45] Kim S. Watarai M. Suzuki H. Makino S. Kodama T. Shirahata T. (2004). Lipid raft microdomains mediate class a scavenger receptor-dependent infection of *Brucella* abortus. Microb. Pathog. 37, 11. doi: 10.1016/j.micpath.2004.04.002 15194155

[B46] Kurmanov B. Zincke D. Su W. Hadfield T. L. Aikimbayev A. Karibayev T.. (2022). Assays for identification and differentiation of *Brucella* species: a review. Microorganisms 10, 1584. doi: 10.3390/microorganisms10081584 36014002 PMC9416531

[B47] Lamontagne J. Forest A. Marazzo E. Denis F. Butler H. Michaud J. F.. (2009). Intracellular adaptation of *Brucella* abortus. J. Proteome Res. 8, 1594. doi: 10.1021/pr800978p 19216536 PMC2771391

[B48] Li T. Xu Y. Liu L. Huang M. Wang Z. Tong Z.. (2016). *Brucella* melitensis 16M regulates the effect of AIR domain on inflammatory factors, autophagy, and apoptosis in mouse macrophage through the ROS signaling pathway. PloS One 11, e0167486. doi: 10.1371/journal.pone.0167486 27907115 PMC5132199

[B49] Liu A. C. L. Ma Q. L. (2017). Cloning and prokaryotic expression of protein-omp31 of brucellosis sheep and its effect on microglial cell apoptosis. J. Ningxia Med. Univ. 39, 667.

[B50] Lopez-Goni I. Guzman-Verri C. Manterola L. Sola-Landa A. Moriyon I. Moreno E. (2002). Regulation of *Brucella* virulence by the two-component system BvrR/BvrS. Vet. Microbiol. 90, 329. doi: 10.1016/s0378-1135(02)00218-3 12414153

[B51] Lopez-Santiago R. Sanchez-Argaez A. B. De Alba-Nunez L. G. Baltierra-Uribe S. L. Moreno-Lafont M. C. (2019). Immune response to mucosal *Brucella* infection. Front. Immunol. 10. doi: 10.3389/fimmu.2019.01759 PMC6710357 31481953

[B52] Luo C. Laaja P. (2004). Inhibitors of JAKs/STATs and the kinases: a possible new cluster of drugs. Drug Discovery Today 9, 268. doi: 10.1016/S1359-6446(03)03014-9 15003245

[B53] Marchesini M. I. Morrone Seijo S. M. Guaimas F. F. Comerci D. J. (2016). A T4SS effector targets host cell alpha-enolase contributing to *Brucella* abortus intracellular lifestyle. Front. Cell Infect. Microbiol. 6. doi: 10.3389/fcimb.2016.00153 PMC5110553 27900285

[B54] Martinez-Nunez C. Altamirano-Silva P. Alvarado-Guillen F. Moreno E. Guzman-Verri C. Chaves-Olarte E. (2010). The two-component system BvrR/BvrS regulates the expression of the type IV secretion system VirB in *Brucella* abortus. J. Bacteriol 192, 5603. doi: 10.1128/JB.00567-10 20833814 PMC2953682

[B55] Martirosyan A. Moreno E. Gorvel J. P. (2011). An evolutionary strategy for a stealthy intracellular *Brucella* pathogen. Immunol. Rev. 240, 211. doi: 10.1111/j.1600-065X.2010.00982.x 21349096

[B56] McCullough N. B. Beal G. A. (1951). Growth and manometric studies on carbohydrate utilization of *Brucella* . J. Infect. Dis. 89, 266. doi: 10.1093/infdis/89.3.266 14888951

[B57] Micheau O. Tschopp J. (2003). Induction of TNF receptor I-mediated apoptosis *via* two sequential signaling complexes. Cell 114, 181. doi: 10.1016/s0092-8674(03)00521-x 12887920

[B58] Miller C. N. Smith E. P. Cundiff J. A. Knodler L. A. Bailey Blackburn J. Lupashin V.. (2017). A *Brucella* type IV effector targets the COG tethering complex to remodel host secretory traffic and promote intracellular replication. Cell Host Microbe 22, 317. doi: 10.1016/j.chom.2017.07.017 28844886 PMC5599354

[B59] Myeni S. Child R. Ng T. W. Kupko J. J. 3rd Wehrly T. D. Porcella S. F.. (2013). *Brucella* modulates secretory trafficking *via* multiple type IV secretion effector proteins. PloS Pathog. 9, e1003556. doi: 10.1371/journal.ppat.1003556 23950720 PMC3738490

[B60] Olsen S. C. Palmer M. V. (2014). Advancement of knowledge of *Brucella* over the past 50 years. Vet. Pathol. 51, 1076. doi: 10.1177/0300985814540545 24981716

[B61] Pandey A. Lin F. Cabello A. L. da Costa L. F. Feng X. Feng H. Q.. (2018). Activation of host IRE1alpha-dependent signaling axis contributes the intracellular parasitism of *Brucella* melitensis. Front. Cell Infect. Microbiol. 8. doi: 10.3389/fcimb.2018.00103 PMC5919948 29732320

[B62] Pasquali P. Thornton A. M. Vendetti S. Pistoia C. Petrucci P. Tarantino M.. (2010). CD4^+^CD25^+^ T regulatory cells limit effector T cells and favor the progression of brucellosis in BALB/c mice. Microbes Infect. 12, 3. doi: 10.1016/j.micinf.2009.09.005 19772948

[B63] Pei J. Kahl-McDonagh M. Ficht T. A. (2014). *Brucella* dissociation is essential for macrophage egress and bacterial dissemination. Front. Cell Infect. Microbiol. 4. doi: 10.3389/fcimb.2014.00023 PMC3942807 24634889

[B64] Pizarro-Cerda J. Meresse S. Parton R. G. van der Goot G. Sola-Landa A. Lopez-Goni I.. (1998). *Brucella* abortus transits through the autophagic pathway and replicates in the endoplasmic reticulum of nonprofessional phagocytes. Infect. Immun. 66, 5711. doi: 10.1128/IAI.66.12.5711-5724.1998 9826346 PMC108722

[B65] Porte F. Liautard J. P. Kohler S. (1999). Early acidification of phagosomes containing *Brucella* suis is essential for intracellular survival in murine macrophages. Infect. Immun. 67, 4041. doi: 10.1128/IAI.67.8.4041-4047.1999 10417172 PMC96697

[B66] Priem D. van Loo G. Bertrand M. J. M. (2020). A20 and cell death-driven inflammation. Trends Immunol. 41, 421. doi: 10.1016/j.it.2020.03.001 32241683

[B67] Roop R. M. 2nd Gee J. M. Robertson G. T. Richardson J. M. Ng W. L. Winkler M. E. (2003). *Brucella* stationary-phase gene expression and virulence. Annu. Rev. Microbiol. 57, 57. doi: 10.1146/annurev.micro.57.030502.090803 12730323

[B68] Sola-Landa A. Pizarro-Cerda J. Grillo M. J. Moreno E. Moriyon I. Blasco J. M.. (1998). A two-component regulatory system playing a critical role in plant pathogens and endosymbionts is present in *Brucella* abortus and controls cell invasion and virulence. Mol. Microbiol. 29, 125. doi: 10.1046/j.1365-2958.1998.00913.x 9701808

[B69] Spera J. M. Comerci D. J. Ugalde J. E. (2014). *Brucella* alters the immune response in a prpA-dependent manner. Microb. Pathog. 67-68, 8. doi: 10.1016/j.micpath.2014.01.003 24508400 PMC4040946

[B70] Stranahan L. W. Arenas-Gamboa A. M. (2021). When the going gets rough: the significance of *Brucella* lipopolysaccharide phenotype in host-pathogen interactions. Front. Microbiol. 12. doi: 10.3389/fmicb.2021.713157 PMC8319746 34335551

[B71] Sun X. L. Chen B. Y. Zhao H. K. Cheng Y. Y. Zheng M. H. Duan L.. (2016). Gas1 up-regulation is inducible and contributes to cell apoptosis in reactive astrocytes in the substantia nigra of LPS and MPTP models. J. Neuroinflamm. 13, 180. doi: 10.1186/s12974-016-0643-2 PMC4938987 27391369

[B72] Taguchi Y. Imaoka K. Kataoka M. Uda A. Nakatsu D. Horii-Okazaki S.. (2015). Yip1A, a novel host factor for the activation of the IRE1 pathway of the unfolded protein response during *Brucella* infection. PloS Pathog. 11, e1004747. doi: 10.1371/journal.ppat.1004747 25742138 PMC4350842

[B73] Uzureau S. Lemaire J. Delaive E. Dieu M. Gaigneaux A. Raes M.. (2010). Global analysis of quorum sensing targets in the intracellular pathogen *Brucella* melitensis 16 m. J. Proteome Res. 9, 3200. doi: 10.1021/pr100068p 20387905 PMC2880877

[B74] Vallabhapurapu S. Karin M. (2009). Regulation and function of NF-κB transcription factors in the immune system. Annu. Rev. Immunol. 27, 693. doi: 10.1146/annurev.immunol.021908.132641 19302050

[B75] Vereecke L. Beyaert R. van Loo G. (2009). The ubiquitin-editing enzyme A20 (TNFAIP3) is a central regulator of immunopathology. Trends Immunol. 30, 383. doi: 10.1016/j.it.2009.05.007 19643665

[B76] Viadas C. Rodriguez M. C. Sangari F. J. Gorvel J. P. Garcia-Lobo J. M. Lopez-Goni I. (2010). Transcriptome analysis of the *Brucella* abortus BvrR/BvrS two-component regulatory system. PloS One 5, e10216. doi: 10.1371/journal.pone.0010216 20422049 PMC2858072

[B77] Wang F. Hu S. Liu W. Qiao Z. Gao Y. Bu Z. (2011). Deep-sequencing analysis of the mouse transcriptome response to infection with *Brucella* melitensis strains of differing virulence. PloS One 6, e28485. doi: 10.1371/journal.pone.0028485 22216095 PMC3247208

[B78] Wang P. Qiu W. Dudgeon C. Liu H. Huang C. Zambetti G. P.. (2009). PUMA is directly activated by NF-κB and contributes to TNF-alpha-induced apoptosis. Cell Death Differ 16, 1192. doi: 10.1038/cdd.2009.51 19444283 PMC2872087

[B79] Watarai M. Kim S. Erdenebaatar J. Makino S. Horiuchi M. Shirahata T.. (2003). Cellular prion protein promotes *Brucella* infection into macrophages. J. Exp. Med. 198, 5. doi: 10.1084/jem.20021980 12847134 PMC2196088

[B80] Weeks J. N. Galindo C. L. Drake K. L. Adams G. L. Garner H. R. Ficht T. A. (2010). *Brucella* melitensis VjbR and C12-HSL regulons: contributions of the n-dodecanoyl homoserine lactone signaling molecule and LuxR homologue VjbR to gene expression. BMC Microbiol. 10, 167. doi: 10.1186/1471-2180-10-167 20529360 PMC2898763

[B81] Wei P. Cui G. Lu Q. Yang L. Guan Z. Sun W.. (2015). A20 promotes *Brucella* intracellular growth *via* inhibition of macrophage cell death and activation. Vet. Microbiol. 175, 50. doi: 10.1016/j.vetmic.2014.11.006 25433453

[B82] Weiss G. Schaible U. E. (2015). Macrophage defense mechanisms against intracellular bacteria. Immunol. Rev. 264, 182. doi: 10.1111/imr.12266 25703560 PMC4368383

[B83] Wertz I. E. O'Rourke K. M. Zhou H. Eby M. Aravind L. Seshagiri S.. (2004). De-ubiquitination and ubiquitin ligase domains of A20 downregulate NF-κB signalling. Nature 430, 694. doi: 10.1038/nature02794 15258597

[B84] Xiong X. Li B. Zhou Z. Gu G. Li M. Liu J.. (2021). The VirB system plays a crucial role in *Brucella* intracellular infection. Int. J. Mol. Sci. 22, 13637. doi: 10.3390/ijms222413637 34948430 PMC8707931

[B85] Yi J. Wang Y. Deng X. Shao Z. Zhang H. Wang B.. (2018). The role of JAK2/STAT3 signaling pathway regulation in macrophage apoptosis during *Brucella* M5-90 infection. Kafkas. Univ. Vet. Fak. Derg 24, 563. Available at: http://vetdergikafkas.org/uploads/pdf.

[B86] Xiao Y Li M Guo X Zeng H Shuai X Guo J. (2022). Inflammatory mechanism of *Brucella* infection in placental trophoblast cells. Int. J. Mol. Sci. 23, 13417. doi: 10.3390/ijms232113417 36362199 PMC9657658

[B87] Zhang K. Wang H. Guo F. Yuan L. Zhang W. Wang Y.. (2016). OMP31 of *Brucella* melitensis 16M impairs the apoptosis of macrophages triggered by TNF-alpha. Exp. Ther. Med. 12, 2783. doi: 10.3892/etm.2016.3655 27698784 PMC5038375

